# Bilateral Chronic Expanding Hematomas of the Gluteus Maximus Presenting With Gluteal Muscle Contracture: A Case Report

**DOI:** 10.1002/iid3.70336

**Published:** 2026-01-15

**Authors:** Xi‐Qing Pan, Jin‐Hui Liu, Jiang‐Li Zhang, Ya‐Ya Zhang, Jian‐Yong Zhang, Lei Shu, Wei Zhao

**Affiliations:** ^1^ Department of Joint Surgery Third Hospital of Shijiazhuang Shijiazhuang Hebei China

**Keywords:** chronic expanding hematoma, gluteal muscle contracture, gluteus maximus

## Abstract

**Background:**

Chronic expanding hematoma is defined as a progressively enlarging soft tissue mass persisting beyond 1 month, typically resulting from prior trauma or surgical procedures. Although the condition is rare, its occurrence within deep musculature—particularly with bilateral involvement of the gluteus maximus—is exceedingly uncommon. No previously published English‐language reports were identified describing bilateral gluteus maximus hematomas associated with gluteal muscle contracture (GMC).

**Case Report:**

This report describes a 29‐year‐old male presenting with bilateral chronic expanding hematomas involving the gluteus maximus muscles, accompanied by clinical features consistent with GMC.

**Conclusion:**

Chronic expanding hematoma should be considered a potential underlying etiology in individuals presenting with GMC, particularly when involving deep gluteal musculature.

**Abbreviations**
CT: computed tomography
GMC: gluteal muscle contracture
ITB: iliotibial band
MRI: magnetic resonance imaging

## 1 Background

Gluteal muscle contracture (GMC), as the name suggests, is a clinical syndrome characterized by the contracture of gluteal muscles, iliotibial band (ITB), and related fascia, in severe cases hip external rotators and rarely hip joint capsule [1]. Patients with GMC typically present with abducted and externally rotated hip and are unable to bring both knees together when squatting [2]. Regarding the etiology, different possible hypotheses have been put forward, namely; idiopathic [3], genetic [4] or congenital [5], and postnatal or acquired.

Chronic expanding hematoma is characterized by a progressively enlarging soft tissue mass that persists for more than 1 month [6, 7]. This condition is rare, and its pathogenesis remains unclear. Labadie and Glover hypothesized that repeated capillary hemorrhage beneath the fibrous pseudocapsule may contribute to the gradual enlargement of the lesion [8]. In contrast, Friedlander and Bump suggested that chronic expansion may result from an increased osmotic pressure gradient caused by the degradation of hematoma components [9].

## 2 Case Presentation

A 29‐year‐old male presented with a 10‐year history of an inability to perform a full squat while maintaining medial knee contact. His medical history revealed an episode of acute tonsillitis approximately 20 years earlier, during which a daily regimen of intramuscular injections had been administered for about 1 month.

## 3 Physical Examination

**Figure 1:**
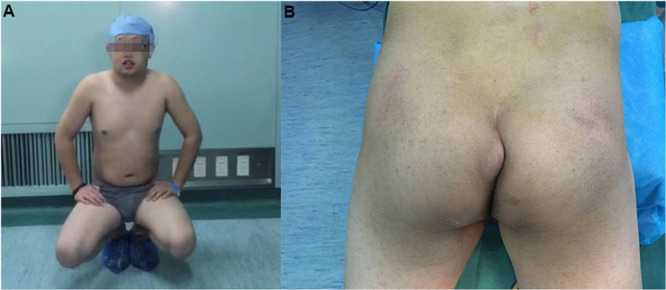
A: Physical examination demonstrated an inability to maintain medial knee contact during a balanced squatting position. B: Swelling observed at the site of previous gluteal intramuscular injections.

Physical examination revealed an inability to maintain medial knee approximation during a balanced squat (Figure 1A). Inspection of the gluteal region revealed bilateral swelling at the sites corresponding to previous intramuscular injections (Figure 1B). Palpation of the affected areas revealed two large, elastic, and fluctuant masses with mild tenderness. There were no signs of localized erythema, increased warmth, or overt inflammation. Preoperative hip joint function (both sides): (1) **Adduction angle:** 5°; (2) **Flexion angle:** 100°; (3) **Internal rotation angle:** 15°; (4) **Function:** Unable to squat with knees together, crossing legs in a double‐lotus position is completely impossible.

## 4 Imageological Examination

**Figure 2:**
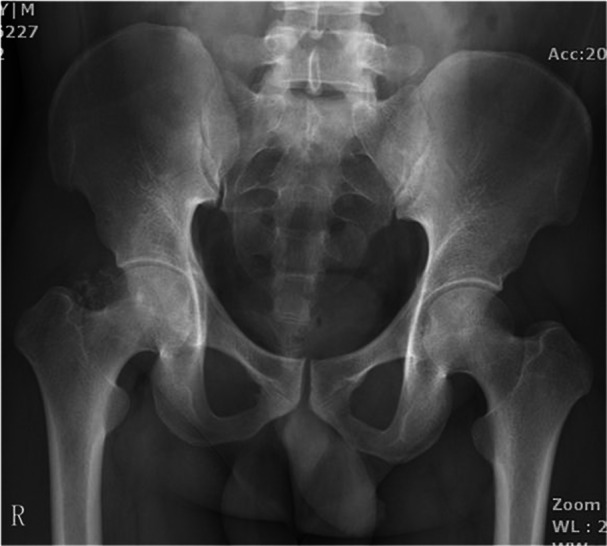
Plain radiograph of the right gluteal region showing irregular contours and scattered soft tissue calcifications. The corresponding radiograph on the left side reveals no evidence of soft tissue calcification or bony lesions.

**Figure 3:**
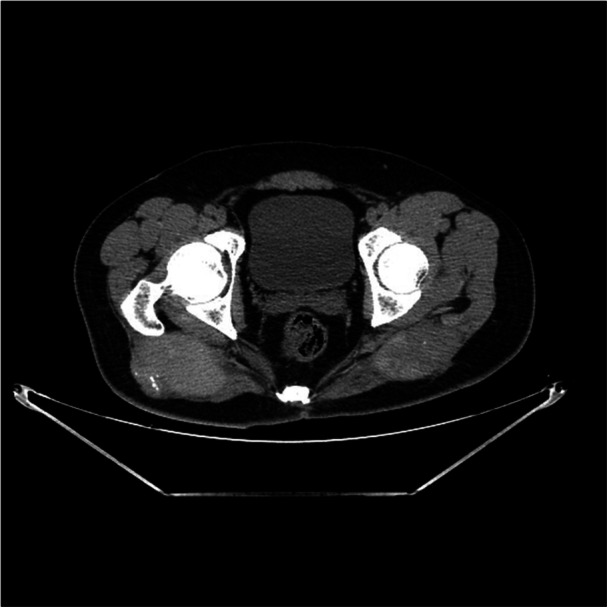
CT demonstrates bilateral enlargement of the gluteus maximus muscles with irregular soft tissue margins. Heterogeneous soft tissue density observed around a lamellar calcification on the right and a punctate calcification on the left.

**Figure 4:**
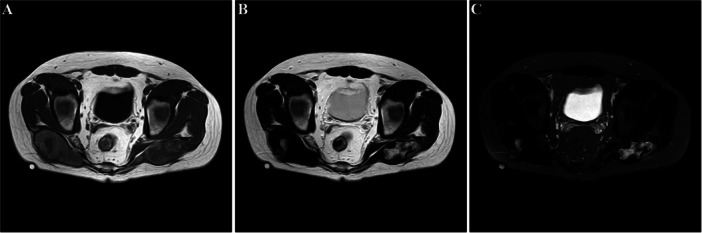
A: T1‐weighted MRI images demonstrate hyperintense signals in the peripheral regions and hypointense signal centrally within the lesions. B: T2‐weighted images reveal hypointensity peripherally and hyperintensity in the central regions. C: Fat‐suppressed T2‐weighted sequences reveal similar findings, with low signal intensity at the periphery and high signal intensity in the central areas of the lesions.

Imaging studies, including plain radiography, computed tomography (CT), and magnetic resonance imaging (MRI), were performed for further evaluation (Figures 2, 3, 4). Plain radiography of the right gluteal region demonstrated irregular cortical outlines with scattered soft tissue calcifications. The left gluteal region did not demonstrate soft tissue calcification or bony abnormalities (Figure 2). CT imaging revealed bilateral enlargement of the gluteus maximus muscles with irregular soft tissue margins. Heterogeneous soft tissue density was noted around lamellar calcification on the right side and punctate calcification on the left (Figure 3). MRI revealed two large cystic lesions involving the bilateral gluteus maximus muscles (Figure 4A–C). On T1‐weighted images, the lesions demonstrated hyperintensity in the peripheral zones and hypointensity centrally (Figure 4A). T2‐weighted images exhibited the inverse pattern, with hypointensity peripherally and hyperintensity centrally (Figure 4B). Fat‐suppressed T2‐weighted sequences similarly showed low signal intensity at the periphery and high signal intensity in the central areas of the lesions (Figure 4C). Both lesions were encapsulated with thin, well‐defined borders.

## 5 Operation

Following biopsy confirmation, surgical excision of the bilateral gluteal masses was performed. Based on the preoperative MRI images, the projected extent of the hematoma and the incision line were marked on the skin with a sterile pen. A single full‐thickness skin incision was made with a scalpel. Subcutaneous adipose tissue was sequentially divided with electrocautery, which simultaneously coagulated small vessels and achieved hemostasis. The fascia was elevated with forceps and incised carefully with either a scalpel or electrocautery. Upon opening the fascia, the gluteus maximus muscle fibers, which exhibited a consistent orientation, were visualized. A plane was gently initiated along the fiber direction with the tip of a vascular clamp, and the hematoma was bluntly dissected. Sharp dissection was performed with electrocautery within the pseudocapsular plane 1–2 mm external to the capsule. As dissection progressed, the intact contour of the hematoma was gradually revealed. Small vessels encountered were precisely coagulated with electrocautery or ligated with silk sutures. Dissection was maintained in close apposition to the outer capsular wall, thereby displacing surrounding normal tissues. Once all capsular attachments were divided, the entire chronic hematoma was removed en bloc from the wound cavity.

**Figure 5:**
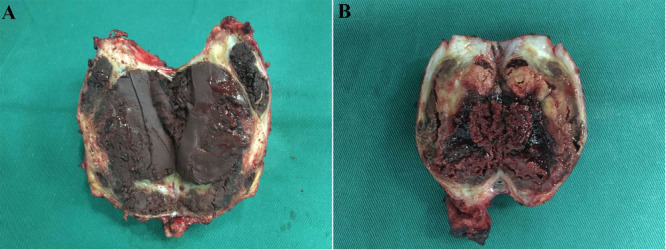
Intraoperative findings revealed extensive bilateral involvement of the gluteus maximus muscles, with the presence of two large, irregularly shaped, thick‐walled cystic lesions. The edges of the excised lesions appeared hyperemic. The internal surfaces of the cysts were uneven, containing granulomatous tissue and organized, chronically retained blood clots. The dimensions of the lesions measured 7.5 × 5.0 × 3.5 cm on the left side (A) and 10.5 × 11.0 × 5.5 cm on the right side (B).

Intraoperative findings revealed extensive bilateral involvement of the gluteus maximus muscles, with the presence of two large, irregularly shaped, thick‐walled cystic lesions. The edges of the excised lesions appeared hyperemic. The internal surfaces of the cysts were uneven, containing granulomatous tissue and organized, chronically retained blood clots (Figures 5A,5B).

## 6 Histopathological Examination

**Figure 6:**
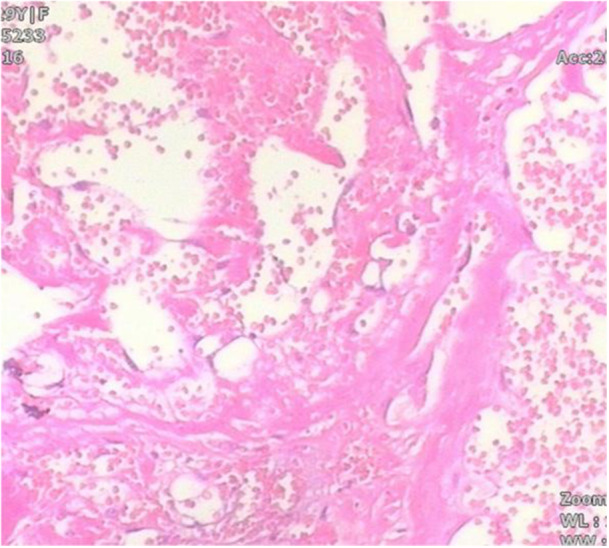
Microscopic examination reveals cystic lesion walls composed of dense fibrous connective tissue exhibiting hyaline degeneration. The inner surface demonstrates limited capillary ingrowth and presence of blood clots. Extensive necrotic tissue observed in the central regions of the lesions.

Histopathological examination of the resected specimens demonstrated organized dark red hematoma material encased within dense, gray‐white fibrous tissue. The dimensions of the lesions measured 7.5 × 5.0 × 3.5cm on the left side and 10.5 × 11.0 × 5.5cm on the right side. Microscopic examination demonstrated that the cystic lesion walls were composed of dense fibrous connective tissue exhibiting areas of hyaline degeneration. The inner surfaces of the cystic walls contained organized blood clots and proliferating capillaries extending inward. Extensive necrotic tissue was observed centrally within the lesions (Figure 6). Immunohistochemical analysis revealed strong positive staining for CD34, CD31, and D2‐40 in the vascular endothelium. The Ki‐67 proliferation index exceeded 10%, indicating moderate cellular proliferative activity.

## 7 Postoperative Evaluation

At the 8‐week postoperative evaluation, there was a marked reduction in hip abduction and external rotation deformity observed during squatting. Functional improvement in both squatting and sitting movements was also noted. Postoperative hip joint function (both sides): (1) **Adduction angle:** 25° (reaching the normal range); (2) **Flexion angle:** 135° (close to the upper limit of normal range); (3) **Internal rotation angle:** 35° (within the normal range); (4) **Function:** Able to perform natural full squat with knees together, cross legs freely, normal gait restored, no discomfort.

## 8 Discussion

The clinical presentation of this patients was consistent with features characteristic of GMC. Notably, the localized swelling at prior injection sites had not been recognized. There was a substantial risk of misdiagnosis as a typical case of GMC. In such instances, the omission of appropriate imaging evaluation could lead to diagnostic error and may constitute a lapse in clinical care.

A comprehensive review of the English‐language literature revealed no previously documented cases of chronic expanding hematomas involving the gluteus maximus muscles that mimicked the clinical presentation of GMC. However, the patient's medical history included a prior course of multiple intramuscular injections. Chronic expanding hematoma was then considered a potential underlying etiology.

Chronic expanding hematoma typically contain a mixture of both recent and organized hemorrhagic material, encased within a fibrous pseudocapsule. Additional findings frequently include necrotic debris and regions of liquefactive degeneration [10]. Puncture biopsy can reveal a dense, fibrous outer capsule. Microscopic examination often demonstrates degradation products of erythrocytes, granulation tissue, neovascularization, and infiltrating inflammatory cells [11]. Chronic expanding hematomas may remain clinically indolent for extended periods before undergoing sudden expansion, resembling the behavior observed in chronic subdural hematomas. The underlying pathophysiology remains incompletely understood.

Although a history of trauma has been documented in several cases of chronic expanding hematomas, it is not universally present [12, 13, 14]. Sakamoto and Matsuda reported cases of chronic expanding hematomas developing between 24 and 45 years after thoracoplasty [15]. The underlying pathophysiological mechanism has been attributed to inflammation triggered by hemolysis, wherein cytolytic byproducts stimulate a fibroblastic response. The resulting inflammatory milieu increases vascular permeability, facilitating capillary hemorrhage into the granulation tissue beneath the pseudomembranous capsule, thereby promoting progressive hematoma expansion [16].

Differentiating chronic expanding hematomas from malignant neoplasms may be challenging due to overlapping features such as gradual lesion growth and, in some cases, bone erosion [17]. MRI serves as a valuable diagnostic tool in such cases. Characteristic findings include central signal heterogeneity and peripheral low signal intensity on both T1‐weighted and T2‐weighted sequences, consistent with a mixture of recent and degraded blood products. The presence of a fibrous pseudocapsule with uniform low signal intensity on all sequences further supports a diagnosis of hematoma. The fibrous pseudocapsule may occasionally be mistaken for attenuated cortical bone due to its appearance on imaging.

In this case, the formation of the hematoma was attributed to capillary injury resulting from repeated intramuscular injections. Progressive enlargement over several years is characteristic of chronic expanding hematomas; however, the 20‐year duration documented here represents the longest interval reported in English‐language literature.

Differential diagnosis based on radiographic imaging should include hemangioma, sarcoma, sclerofibroma, and malignant fibrous histiocytoma. Arterial hemangioma typically demonstrates low signal intensity on both T1‐weighted and T2‐weighted sequences. In contrast, venous hemangioma often presents with low peripheral signal intensity and high central signal intensity on T2‐weighted images, without associated soft tissue calcifications. Sarcomas generally display a homogenous signal pattern, characterized by high signal intensity on T2‐weighted images and low signal intensity on T1‐weighted images. On T2‐weighted images, malignant neoplasms may present as protruding or irregularly shaped masses with slightly increased signal intensity. Although malignant fibrous histiocytoma rarely involves bilateral gluteal musculature, it typically presents with poorly defined margins and a lack of intramuscular confinement.

Immunohistochemical analysis in this case demonstrated a Ki‐67 proliferation index of approximately 10%, with no histological features suggestive of malignancy, supporting the diagnosis of a benign lesion. Positive staining for CD34, CD31, and D2‐40 indicated vascular endothelial origin; however, differentiation from vascular malformations remained essential. Most vascular malformations lack a defined capsule and are composed predominantly of proliferative vascular channels. In contrast, the lesion described in this case exhibited a fibrous capsule with hyaline degeneration, central necrosis, and limited capillary ingrowth—features more consistent with chronic hematoma than vascular malformation.

## 9 Conclusion

This case demonstrated an association between a history of repeated intramuscular injections and the subsequent development of chronic expanding hematomas, which progressively extended into the gluteus maximus muscle. The progressive enlargement of these hematomas appeared to be influenced by both the anatomical structure and functional dynamics of the involved musculature. Additionally, the clinical presentation shared overlapping features with GMC, highlighting the need for careful differential diagnosis. This case emphasizes the importance of comprehensive clinical evaluation and detailed medical history‐taking in identifying atypical presentations. Failure to recognize localized swelling and characteristic imaging features may lead to diagnostic oversight and inappropriate management.

## Author Contributions

Conception and design of the research: **Jin‐Hui Liu**, **Xi‐Qing Pan**. Acquisition of data: **Ya‐Ya Zhang**, **Jian‐Yong Zhang**, **Lei Shu**, **Wei Zhao**.Analysis and interpretation of the data: **Jiang‐Li Zhang**. Statistical analysis: **Xi‐Qing Pan**. Obtaining financing: None; Writing of the manuscript: **Xi‐Qing Pan**. Critical revision of the manuscript for intellectual content: **Xi‐Qing Pan**. All authors read and approved the final draft.

## Funding

The authors received no specific funding for this work.

## Ethics Statement

This study was conducted with approval from the Ethics Committee of Third Hospital of Shijiazhuang. This study was conducted in accordance with the declaration of Helsinki. Written informed consent was obtained from all participants.

## Consent

The participant signed a document of informed consent.

## Conflicts of Interest

The authors declare no conflicts of interest.

## AI Statement

We declare that we have not used any any form of artificial intelligence (AI), including generative tools such as ChatGPT or similar technologies, was used during the preparation of our manuscript.

## Data Availability

The datasets used and/or analyzed during the current study are available from the corresponding author upon reasonable request.
